# Lower gut dysbiosis and mortality in acute critical illness: a systematic review and meta-analysis

**DOI:** 10.1186/s40635-022-00486-z

**Published:** 2023-02-03

**Authors:** Tess Evans, Umar Ali, Ryan Anderton, Edward Raby, Laurens Manning, Edward Litton

**Affiliations:** 1 Intensive Care Unit, Fiona Stanley Hospital, South Metropolitan Health Service, WA Health, Perth, Australia; 2 Department of Infectious Diseases, Fiona Stanley Hospital, South Metropolitan Health Service, WA Health, Perth, Australia; 3 School of Medicine, University of Western Australia, Nedlands, Australia; 4 School of Health Sciences, University of Notre Dame Australia (Fremantle), Fremantle, Australia

**Keywords:** Gut, Microbiome, Dysbiosis, Critical illness, Sepsis

## Abstract

**Background:**

The human gastrointestinal tract harbours a complex multi-kingdom community known as the microbiome. *Dysbiosis* refers to its disruption and is reportedly extreme in acute critical illness yet its clinical implications are unresolved. The review systematically evaluates the association between gut dysbiosis and clinical outcomes of patients early in critical illness.

**Methods:**

Following PRISMA guidelines, a prospectively registered search was undertaken of MEDLINE and Cochrane databases for observational studies undertaking metagenomic sequencing of the lower gastrointestinal tract of critically ill adults and children within 72 h of admission. Eligible studies reported an alpha diversity metric and one or more of the primary outcome, in-hospital mortality, or secondary clinical outcomes. After aggregate data were requested, meta-analysis was performed for four studies with in-hospital mortality stratified to high or low Shannon index.

**Results:**

The search identified 26 studies for systematic review and 4 had suitable data for meta-analysis. No effect of alpha diversity was seen on in-hospital mortality after binary transformation of Shannon index (odds ratio 0.52, CI 0.12–4.98, *I*^2^ = 0.64) however certainty of evidence is low. Pathogen dominance and commensal depletion were each more frequently associated with in-hospital mortality, adverse clinical and ecological sequelae, particularly overabundance of *Enterococcus*.

**Conclusions:**

There is a paucity of large, rigorous observational studies in this population. Globally, alpha diversity was dynamically reduced in early ICU admission in adults and children and was not associated with in-hospital mortality. The abundance of taxa such as *Enterococcus* spp. appears to offer greater predictive capacity for important clinical and ecological outcomes.

**Supplementary Information:**

The online version contains supplementary material available at 10.1186/s40635-022-00486-z.

## Background

The lower gastrointestinal tract microbiome is a complex multi-kingdom community of bacteria, archaea, fungi and viruses. In humans, this community is shaped in early life, but modified by age, environment, diet, disease states and antibiotic administration [1]. Due to the taxonomic complexity of the system, microbiomes are typically characterised using measures of relative abundance of important families or genera, or diversity. Alpha diversity represents the richness (number) and evenness (spread) of species as a summary statistic of a single population, often expressed by Shannon or Simpson's indices (see Additional file 2: Glossary) [2]. Beta diversity describes the absolute or relative taxonomic overlap between clinical samples over time, between individuals or between body sites (biogeography) [3]. A typical healthy microbiome sees high alpha diversity with a predominance of *Firmicutes* and *Bifidobacteria*. In this state, the microbiome’s impact as a modulator of metabolism and immune function is thought to be optimised, though the mechanistic links for these processes are only now being elucidated [4]. Disruption of microbiome community structure and metabolic function is termed dysbiosis. Whilst there is no global consensus definition, the prototypical disease state associated with dysbiosis is *Clostridioides difficile* associated diarrhoea which is accompanied by profound loss of alpha diversity with increased relative abundance of *Enterobacteriaceae* and *Enterococcaceae* families [5]. However, dysbiosis is also associated with many other chronic inflammatory, autoimmune and neuropsychiatric conditions [6, 7].

Patients presenting with acute critical illness are at especially high risk of dysbiosis [8, 9]. Co-morbidities, severe metabolic disturbance and the frequent use of antibiotics in intensive care units are thought to drive the disproportionate depletion of commensal organisms and emergence of opportunistic pathogens [10]. Whilst severe dysbiosis in these settings is thought to confer a poorer prognosis, the association between conventionally reported microbiome characteristics and adverse clinical outcomes has not been systematically reported.

In preparation for a large observational study of lower gut microbiome in patients with acute critical illness admitted to a tertiary intensive care unit (ICU) and a trial of salvage faecal microbiota transplantation (FMT) for patients with presumed dysbiosis, we aimed to systematically review publications reporting associations between lower gut dysbiosis by metagenomic techniques and adverse clinical outcomes in critically ill adults or children. We hypothesised that amongst patients who undergo sampling of lower gut microbiota within 72 h of admission with critical illness, lower alpha-diversity will be associated with in-hospital mortality and certain pathogenic genera (*Enterococcus*, *Streptococcus*, *Pseudomonas*) will exhibit greater relative abundance.

## Methods

### Search strategy and selection criteria

This systematic review was prospectively registered (PROSPERO CRD 42,022,281,530) and conducted in accordance with Preferred Reporting Items for Systematic Reviews and Meta-analyses (PRISMA) guidelines. The inclusion criteria included prospective or retrospective observational studies examining critically ill adults or children admitted to an intensive care unit who underwent microbiome analyses by molecular methods, including 16S rRNA and shotgun metagenomic sequencing, where Shannon index or other alpha diversity index was reported as exposure variable. Neonates were excluded given known dynamic differences in microbiome composition. To capture acute critical illness, we restricted the analysis to studies reporting a majority of rectal or stool sampling within 72 h of ICU admission and only samples within this timeframe were included in meta-analysis. Strong concordance of stool and swab samples has been demonstrated [11–13] and results were therefore pooled.

Representing the weighted geometric mean of proportional abundances of taxa within a sample, Shannon index was defined as high when greater than or equal to 3, and low when less than 3 based upon the mean value of included studies. Studies reporting any of the primary or secondary outcome variables described below were eligible for inclusion. Operational Taxonomic Units (OTUs) which were significantly enriched or depleted as determined by the study authors were recorded. Abstract-only, non-English language publications and culture-based studies were excluded.

### Outcomes

**Fig. 1:**
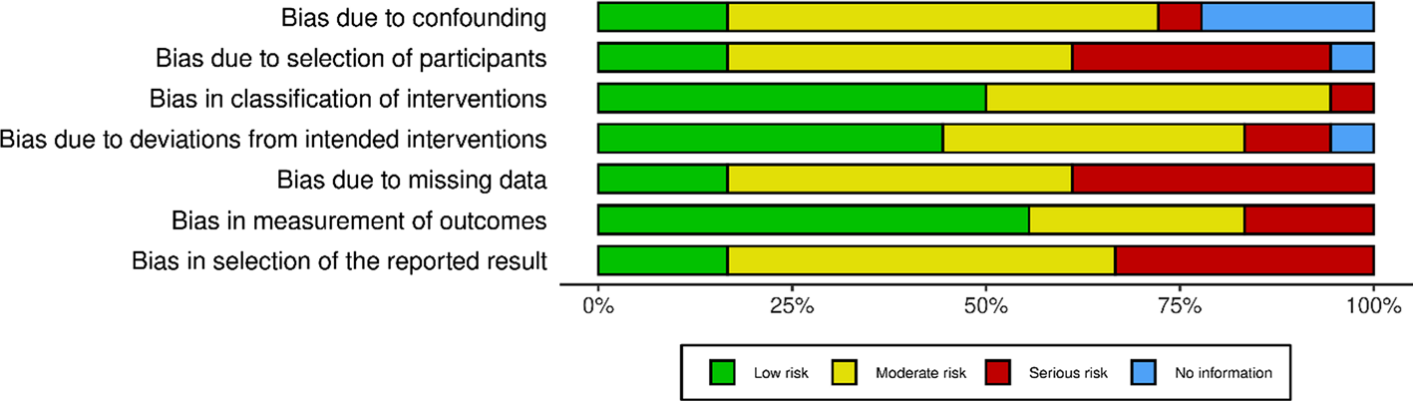
Cochrane Risk of Bias for non-interventional studies (ROBINS-II) included in systematic review (*n* = 26). Breakdown per study demonstrated in Additional file 1: Fig. S6

The primary outcome was all-cause in-hospital mortality at day 28, censored at hospital discharge. Secondary outcomes included (i) ICU length of stay, (ii) overall hospital length of stay, (iii) mortality at longest follow-up censored to day 180), (iv) duration of mechanical ventilation (MV) and (v) incidence of infection. Pre-specified subgroups of interest included (i) presentations with sepsis (as defined by the study), (ii) adults (compared with children), (iii) surgical (compared with medical admissions), (iv) severe illness (compared with non-severe illness), (v) patients receiving prior antibiotic therapy and (vi) rectal sampling (compared with stool). Risk of bias assessment was performed using the Cochrane tool for non-interventional studies (ROBINS-I) tool [14] (see Fig. 1).

### Study selection

MEDLINE and The Cochrane Central Register of Controlled Trials were searched for relevant observational studies with subsequent manual reference search. The search was limited to articles published after 2011 given the recent advent of molecular sequencing methods. Where outcomes were recorded, but not presented suitably for aggregate analysis, data were directly requested from corresponding authors.

The search strategy included terms related to gut microbiome and critical illness:

*(("critically ill" OR "critical illness" OR "intensive care")) AND ((gut) OR (gastrointestinal) OR (intestinal)) AND ((microbiome) OR (microbiota) OR (pathobiome) OR (dysbiosis) OR (flora)).*

Two authors (T.E. and U.A.) performed the pre-defined literature search, reviewed texts and extracted data independently. Studies were eligible for inclusion in the systematic review where they met all inclusion criteria and none of the exclusion criteria. Discordance was adjudicated by a third reviewer (E.L.).

### Data synthesis

For normally distributed continuous data, the mean and standard deviation was used. Where a median and interquartile range (IQR) were reported, the mean and standard deviation (SD) were imputed using validated techniques [15]. An odds ratio with a 95% confidence interval was calculated for binary data. A *P*-value < 0.05 was considered significant. The effects of age and gender balance on the association between the exposure and the primary outcome were assessed using meta-regression if there is adequate sample size. Heterogeneity was reported using the *I*^2^ which describes total variation across studies not due to sampling error. Treatment effect was determined using DerSimonian and Laird random effects model with Egger’s test [16]. Stata was used for all analyses [17].

## Results

**Fig. 2:**
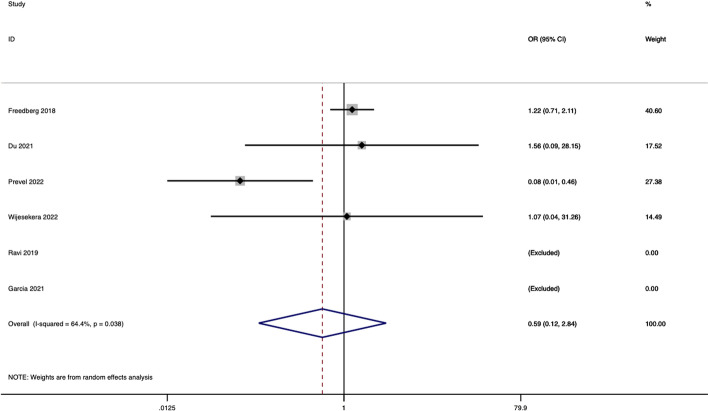
Forest plot displaying aggregate meta-analysis of binary odds ratio of 28-day all-cause mortality in high versus low alpha diversity groups, represented by Shannon index more than 3 or less than 2.99, respectively

There were 26 studies eligible for systematic review of which 7 provided moderate certainty evidence [18–24]. Alpha diversity was significantly predictive of in-hospital mortality in only one [25]. In contrast, pathogen dominance (6 studies) and commensal loss (2 studies) appear to be more highly predictive of in-hospital mortality among other adverse outcomes in adults. In a meta-analysis only 4 studies contributed data, leading to low certainty in the finding of no association between low alpha diversity and in-hospital mortality (odds ratio 0.52, CI 0.12–4.98, *I*^2^ = 0.64, Fig. 2).

### Description of included studies

**Fig. 3:**
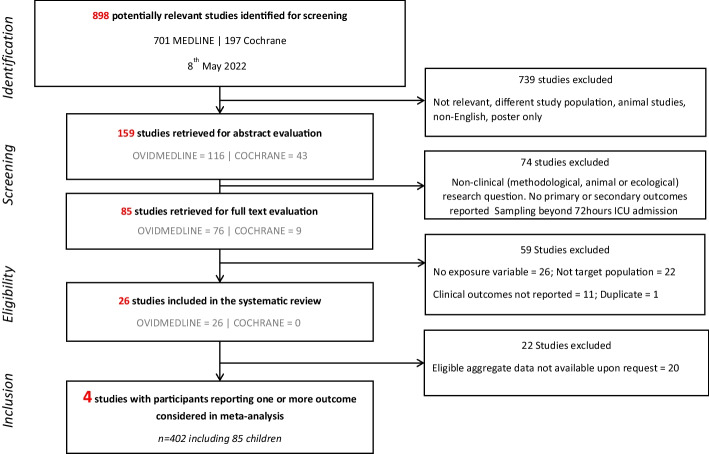
PRISMA flow diagram delineating study selection (*n* = 26 for systematic review, *n* = 4 for meta-analysis)

The PRISMA flow diagram is shown (Fig. 3). A total of 898 studies were screened and 26 studies which reported a total of 1591 patients (142 children and 1503 adults) were included in the systematic review. Most studies were of adults (91.3% of total participants). The median (IQR) age in adult and paediatric studies was 57.3 (52.9–63.8) years and 3.5 years (3.2–4.1), years, respectively. All were prospective observational studies published between 2014 and April 2022 (10, 18, 19, 21–24, 26–39) ranging from 9 to 301 participants (See Additional file 1: Table S1). The majority were single centre (*n* = 20) and half (*n* = 13) were mixed medical and surgical ICUs (Table 1). The overall mean (SD) day 28 all-cause in-hospital mortality was 26.4% (15.9) and ranged between 7–48 and 3–9% in adult and child populations, respectively (Table 2). The overall assessment of risk of bias was high in participant selection, missing data and reporting domains, and moderate in the remainder (Fig. 1).

**Table 1:** Characteristics of cohorts included in systematic review

First author	Year of publication	Country	Number of participants	Number and type of centres			Sampling	Subspeciality or diagnostic group
Zaborin	2014	USA	14	1	Mixed	Adult	Cross-sectional	
Rogers	2016	USA	37	1	Mixed	Paediatric	Cross-sectional	
Yeh	2016	USA	32	1	Surgical	Adult	Longitudinal	
McDonald	2016	USA, Canada	115	5	Mixed	Adult	Longitudinal	
Howard	2017	USA	12	1	Surgical	Adult	Longitudinal	Trauma
Lankelma	2017	Netherlands	34	1	Mixed	Adult	Cross-sectional	
Lamarche	2018	Canada	34	2	Mixed	Adult	Cross-sectional	
Wan	2018	China	15	1	Medical	Adult	Cross-sectional	
**Freedberg**	**2018**	**USA**	**301**	**1**	**Medical**	**Adult**	**Cross-sectional**	
Aardema	2018	Netherlands	97	1	Surgical	Adult	Longitudinal	Cardiosurgical
Bansal	2018	Canada	9	1	Mixed	Adult	Longitudinal	
**Wijeyesekera**	**2019**	**UK**	**60**	**3**	**Mixed**	**Paediatric**	**Longitudinal**	**Sepsis**
Xu	2019	China	98	1	Surgical	Adult	Longitudinal	Neurosurgical
**Ravi**	**2019**	**England**	**24**	**1**	**Mixed**	**Adult**	**Longitudinal**	
Liu	2020	China	64	1	Mixed	Adult	Cross-sectional	Sepsis
Ojima	2020	Japan	71	1	Mixed	Adult	Longitudinal	
Burmeister	2020	USA	67	1	Surgical	Adult	Cross-sectional	Trauma
Fontaine	2020	France	31	1	Medical	Adult	Longitudinal	
Chernevskaya	2020	Russia	18	2	Surgical	Adult	Longitudinal	Neurosurgical; nosocomial pneumonia
Agudelo-Ochoa	2020	Columbia	155	5	Medical	Adult	Longitudinal	Sepsis
**Du**	**2021**	**China**	**25**	**1**	**Medical**	**Paediatric**	**Cross-sectional**	**Sepsis**
**Garcia**	**2021**	**Spain**	**62**	**1**	**Medical**	**Adult**	**Longitudinal**	**Liver Failure**
Liu	2021	China	20	1	Medical	Paediatric	Cross-sectional	
Wei	2021	China	61	1	Medical	Adult	Cross-sectional	
Kuo	2021	USA	78	1	Mixed	Adult	Longitudinal	
**Prevel**	**2022**	**France**	**57**	**1**	**Medical**	**Adult**	**Longitudinal**	

Studies attempted for inclusion in meta-analysis are highlighted in bold

**Table 2:** Clinical and infection-related outcomes of study cohorts included in systematic review

First author	ICU LOS mean (sd)	Hospital LOS mean (sd)	28-day mortality	Mortality at longest follow-up; days	Proportion with sepsis (%)	Septic shock mortality (%)	Antibiotic prior to sampling (%)	Antibiotic at any time (%)	Cumulative incidence HAI
Zaborin			3/10(30%)				100	100	
Rogers	9.64 (6.40)		1/38(3%)					89	
Yeh			3/10 (30%)						25
McDonald	17.3 (13.7)		24/115 (21%)		7.0%		100	100	
Howard			2/12 (17%)				0	25	
Lankelma	10.9 (12.3)*	45.3(70.8)*	11/34 (32%)	0.74	74%	15%	100	100	
Lamarche	13.7 (8.97)	45.3(60.7)	12/34 (35%)		24%	15%			
Wan			4/11(27%)	45.5%; various#	100%		100	100	
**Freedberg**			**76/301 (25%)**						**41**
Aardema	1.53 (1.09)*	10.5(4.28)*	7/96 (7%)				100	100	
Bansal			3/12 25%)		11%				
**Wijeyesekera**			**4/60 (7%)**		**27%**		**100**	**100**	
Xu	15.28 (14.4)		23/98 (23%)	31%; 180					
**Ravi**	**23.2(15.0)**		**4/24 (17%)**		**100%**	**17%**	**42**	**88**	
Liu			20/64 (31%)		100%	31%	100	100	
Ojima	32.1 (49.5)*		21/71 (30%)	34%; 56	15%	55%	90		
Burmeister	15.2 (30.0)*	17.4(13.2)*	8/67(12%)				4		43
Fontaine			15/31 (48%)				55		
Chernevskaya			2/18 (13%)						
Agudelo-Ochoa	7.86(11.7)				46%	26%			
**Du**			**10/25 (40%)**		**100%**	**60%**		**100**	
**Garcia**	11.6(14.5)*	38.7(53.6)*	**18/62(29%)**	**29%; 180**				**100**	**73**
Liu					100%		100	100	
Wei	26.6(43.7)		12/49(20%)		8.2%	60%	0		
Kuo			7/78(9%)						
**Prevel**			**13/57(23%)**		**35%**	**35%**			

Studies included in meta-analysis are highlighted in bold. *Imputed mean (sd) from median with IQR or range using Smith et al. 2016. #censored to hospital discharge. *HAI*  hospital acquired infection

**Table 3:** The microbiome of the critically ill: future research directions

Theme	Approach or concept	Suggestion or example
Literature gaps, data sharing and reporting consensus	Systematic review and meta-analysis	– Chronic critical illness – Loss of biogeography – Utility of serum or faecal metabolites – Microbiome reconstitution – Post-intensive care syndrome and long-term follow-up – Vulnerable diagnostic groups such as solid-organ transplant – The sepsis spectrum and role of anti-microbials – Extremes of age
	Comparability of studies	– Standardisation of microbiome terms – Prepublication of statistical analysis plan – Uniformity of reported outcomes (see STORMS) – Harmonisation of post-processing with testing of robustness and/or validated adjustments based on methodological differences
	Open-access biorepositories	– Publishing to existing repositories or digital platforms (such as American Gut Project) – Mandatory sharing of relevant code (such as github) – Expansion of creative commons licencing
Benchwork models	In vitro	– Organoid models such as SHIME seeks to duplicate ultra-low diversity conditions which could facilitate ‘matching’ of candidate interventions – Cell culture approaches
	Animal models	– *Use of C. elegans* 2-member community model with organisms derived from ICU patients (Zaborin et al.) to probe destructive versus protective signals for phenotype switching
	Systems biology	– Complementary approaches stress-tested in other fields such as soil and forensic science may elucidate relationships which prevent or correct dysbiosis, versus maintenance of a maladaptive steady state
Exploratory	Abstract influences, surrogate markers, extrapolation from healthy populations	– Adjusting for time of day of sampling, patient mobility, and bed location – Identification of bedside urinary or faecal surrogate markers for dysbiosis as seen in Kuo et al. (2021) Testing of robust relationships seen in healthy individuals may unravel risk factors for mortality in critical illness, e.g. *Christensenellaceae* shows strongest association to healthy body mass index and secondary bile acid formation (Waters and Ley, 2019) yet conferred *poorer* prognosis in three ICU studies—raising the question of whether this organism may play a role in the ICU obesity paradox (Sakr et al. 2015)
Clinically occult subgroups	Enterotypes and endotypes	– Clustering across genotype to phenotype levels has been attempted with utility in both prognostication and mechanism assembly – *Enterotypes* are based upon patterns in gut microbiome structure have been found to improve performance of traditional scores in predicting diagnosis (Gu et al.), mortality [33] and surgical complications (Schmitt et al. 2019) – *Endotypes* represent a meaningful, synergistic synthesis of biochemical and physiologic biomarkers and represent an emerging science in this population
Novel clinical trials	Embedded, adaptive, multifaceted designs	– Large multicentre embedded observational studies generalisable to the ICU population are crucial first step – Adequately powered adaptive trials which integrate existing therapies such as artificial feeding with safe candidate therapies such as pre- pro- or syn-biotics – Synchronous metagenomic profiling or enterotype prediction may underlie these precision medicine trials in the short to medium term and incorporate lessons learned from FMT and other vulnerable cohorts such as those with active Crohn’s disease – Later trial phases dedicated to synthetic formulation SER-109, antimicrobial peptides, competitive inhibition approaches such as non-toxigenic *Clostridium difficile* strains and bacteriophage formulations

The most common sample for analysis was stool (*n* = 17), with rectal swabs (*n* = 4), both (n = 3) and examination glove samples (*n* = 2) less commonly used. Rectal swab cohorts saw far greater participation and sampling adherence [11, 23]. For measures of alpha-diversity the Shannon index was the most frequently reported (*n* = 18 studies), followed by Chao richness (*n* = 6)[40], (inverse) Simpsons (*n* = 5)[41] and Pielou evenness (*n* = 2)[42] indices (see Additional file 1: Figs. S1, S2). Beta diversity was reported in most (*n* = 21). Relative abundance was reported in all studies. Design and methodological differences are summarised in Additional file 1: Table S2 and Fig. S3.

Reduced alpha diversity in acute critical illness was reported in 25 of the 26 systematic review studies. The mean (SD) Shannon index within 72 h of admission reported in 9 studies was 3.58 (1.30), lower than in control groups. The lowest mean alpha diversity (Shannon index) in any study, 2.03 (0.71). Alpha diversity declined over time in all 16 longitudinal studies of adults or children (see Additional file 1: Figs. S1, S2). Moderate changes in the first 24–48 h of ICU admission typically became marked by 72 h (exemplified by [36] including those with elective patient load [24]). No relationship between alpha diversity and admission diagnosis was found in 5 of 6 evaluating studies [29, 35]. No associations were observed with artificial feeding patterns or bowel habit [43]. Reduced early alpha diversity after antibiotic exposure was evident in eight studies [18–22, 24, 28, 43], whereas two reported no difference [34, 35]. One study reported a lower Shannon index in non-survivors compared with survivors [25].

### Meta-analysis of alpha diversity and its association with mortality

Although six studies had appropriate data, two studies [20, 43] required exclusion due to two or more categories without mortality events. Meta-analysis of four studies (*n* = 402 including 85 children) found no significant relationship between high versus low alpha diversity and odds ratio of in-hospital mortality at day 28 (OR 0.59, CI 0.12–4.98), censored to hospital discharge. Heterogeneity was seen in study population, sample type, methodology, and post-processing, which was significant between studies (*I*^2^ value was 0.64, *p* = 0.038). The prespecified primary outcome of in-hospital mortality at day 28 or censored to hospital discharge was reported in 24 of 26 studies yet was infrequently categorised by diversity. Authors were contacted, however data were insufficient for calculation of other outcomes and subgroup comparison.

### Outcome and pathogen type

All studies reported taxon abundance measures. *Enterococcus* genus was enriched in 20 of 26 studies (77%). Composition of up to 70% (29), 99.9% (28), 97.9% (31) and 99% (43) in certain individuals was seen.

*Enterococcus spp.* Abundance was associated with in-hospital mortality in four of the included studies [11, 19, 26]. Relative abundance was associated with other adverse outcomes including risk of infection [20, 26], elevated inflammatory markers [33] and ICU length of stay [26]. Additional file 1: Table S3 shows overabundance of suspected and confirmed MDR organisms reported by 14 and 3 studies, respectively. In ecological terms, Enteroccocal dominance was associated with significant loss of microbiome richness and diversity [28, 31]. Fontaine and colleagues found colonisation by vancomycin resistant *Enterococcus* (VRE) but not MDR Gram-negative organisms reduced both richness and evenness [37]. Impact of antibiotic exposure could not be evaluated systematically as only seven studies included drug-specific information.

Commensal organisms may oppose pathogen impact. The only singular commensal taxon to predict survival, Wei et al*.*, showed loss of *Bifidobacterium* was associated ICU mortality (*p* = 0.031) [22]. Together with others—*Blautia* (various species) *Faecalibacterium*, *Collinsella* and *Streptococcus—Bifidobacterium* abundance was associated with survival in one other study [25]. Proportional increase in *Actinobacteria* was shown to interact favourably with SOFA score [18]. Wan reported this phylum was associated with beneficial metabolites and could be selected for (or against) by certain antibiotics. *Prevotella* abundance was associated with non-sepsis within a mixed ICU population [19]. Family X1 and *Prevotellaceae* more abundant in patients who did not acquire MDR organisms [20]. Retention of commensal taxa were associated with reduced risk of carbapenem-resistant *Pseudomonas aeruginosa* acquisition, whereas loss of butyrate producers associated with pathogen overgrowth [24].

### Variability

Beta diversity estimation of interindividual variability of gut microbiome was increased in 20/21 studies reporting this (see Additional file 1: Fig. S4). Longitudinal intra-individual variability was also high in each of 4 reporting studies. Rapid substitution of dominant species was commonly observed only within critically ill populations [31], even at phylum level [18].

There is loss of microbiome distinction between body sites over time. Four studies nested multi-day sampling of forehead/antecubital skin, nares, nasogastric or endotracheal aspirates [23, 31, 35]. Initial biogeography was depleted in richness and evenness with convergence seen in both adults and children. Yeh et al*.* noted commonality of dominant pathogens in distant body sites [23].

### Sepsis subgroup

Infection and sepsis were the most frequently reported subgroups. Hospital-acquired infections were seen in a mean 45.5% of patients of the four reporting studies [20, 23, 26, 39]. Wan [30], Ravi [43], Liu [32], and Du et al*.* [21] recruited only sepsis or septic shock patients. A higher mortality rate was seen among sepsis patients ranging from 17 to 60% in all but two reporting studies which were those employing selective digestive decontamination [34, 35] (Table 2).

Alpha diversity further decreased in sepsis relative to both non-sepsis patients and healthy controls where evaluated [19, 21, 30, 34, 35]. Dysbiosis onset may be delayed: no initial change in alpha diversity was seen in a cohort which later clustered to both sepsis status and source [34]. A longitudinal model noted that sepsis exerted an independent effect on microbiome structure, alongside novel parameters of housing and ICU site [19].

Enrichment of sepsis-specific taxa was apparent, especially *Proteobacteria*. Ojima et al*.* reported higher relative abundance of this genus than other categories [18], and sixfold more frequent in sepsis patients elsewhere [30]. Lankelma et al*.* reported increased *Proteobacteria* within abdominal sepsis only [34]. Du et al*.* noted *Moraxellaceae* was enriched (frequencies are given in Additional file 1: Fig. S6) [21].

## Box 1. Summary of results

**Table:** 

There are universal, clinically meaningful patterns of early gut dysbiosis in critically ill adults and children (see Box 2)
Early pathogen dominance and commensal depletion can predict mortality where meta-analysis of alpha diversity did not in adults
Lack of methodology and nomenclature standardisation, small sample size, and inadequate reporting confer low certainty in meta-analysis result

## Discussion

Although alpha diversity was globally and markedly reduced by 72 h of ICU admission, it did not predict all-cause mortality at day 28 by meta-analysis, in line with 25 of 26 studies in the systematic review [10, 18–35, 37–39, 43, 44]. Confidence in this result is constrained by data availability for meta-analysis, the overall sample size, heterogeneity and moderately high risk of bias.

The novel finding of this systematic review is that overabundance and depletion of selected pathogenic and commensal taxa, respectively, are more frequently predictive of mortality and other clinical outcomes in adults. Alpha diversity metrics do not acknowledge emergence of opportunists, disproportionate loss of obligate anaerobes, infrequent or rare species or detection of syntrophic relationships [45–47]. It is plausible these traditional ecological measures of richness and evenness are too ‘blunt’ to show meaningful compositional change and predict outcomes in acute critical illness.

Whilst interactions between organisms across kingdoms may be best observed in ultra-low diversity conditions, they are also not captured by alpha diversity or common sequencing approaches [6]. 16S rRNA amplification was the primary focus of 24 studies yet does not represent fungal species commonly observed by culture, 18S/ITS and whole genome methods. Prevalence studies report *Candida spp.* in up to 80% of patients 1 week into ICU stay [48]. Ravi noted ESKAPE group and *C. albicans* constituted up to 75% of some microbiomes [43] similar to the pairings of a single bacterial OTU with *C. albicans* seen by Zaborin [28]. Dynamic interplay with fastidious bacterial species may govern the behaviour of *Candida spp.* as an invader, bystander or benefactor given clinical infection is only observed in 5% of those colonised [48] and fungi can recapitulate commensal bacterial functions [49].

Robust and replicable disease-specific patterns were rare [35]. It is plausible that pathogen expansion is largely patient-specific with determination of invasive behaviour by interdependent metabolic, ecological and pharmacologic factors, a hypothesis supported by animal studies [26, 50].

In this regard, antibiotic therapy was seen to be associated with reduced alpha diversity [18–22, 24, 28, 43], particularly carbapenems [38]. Ojima et al. noted the proportion of phylum *Actinobacteria* was severely reduced by this class [18] and was specifically associated with *Pseudomonas* abundance (*p* < 0.01) [19]. Antibiotic usage beyond ICU discharge correlated with sustained loss of alpha diversity in short stay cardiothoracic patients [24].

Antibiotic duration correlated to *Acinetobacter spp.* and *Staphylococcus aureus* abundance [21]. Exposure to more than 6 antibiotics also correlated to development of MDR [28]. Emergence of typical MDR organisms were reported in 17 of 26 studies (see Additional file 1: Fig. S5). Garcia et al*.* noted a median 8-day stay in ICU before acquisition of resistance if this was to occur [20], the same timeframe where diversity is most depleted [51] and where recovery of commensals began, if at all [18]. Circumstantially, these observations suggest day 7–8 in ICU and/or ICU discharge may be imperative junctures for antibiotic rationalisation [18, 20, 24].

### There are universal features of early gut dysbiosis amongst the critically ill

Dysbiosis is complex and dynamic within critically ill patients, yet there were a series of commonalities. These patterns were consistent between studies of adults and children, noting paediatric cohorts were smaller. Taxonomic clustering shows separation of microbiota from that of endemic volunteers, distant healthy controls and other hospitalised patients [52, 53]. Loss of biogeographic regionalisation across distant body sites was distinctive of critical illness. Inter- and intra-individual variability seen amongst ICU patients is much greater than that seen in healthy controls [10]. Each of these changes poorly explained by diagnosis, syndrome, or univariate clinical parameters and are amplified in patients with sepsis [21]. A minor contribution is made by demographic factors and disease severity [19] and heritability may be minimal [45], rendering the microbiome an attractive target for ICU interventions.

Regarding composition, richness and evenness is progressively depleted over the first 72 h of ICU stay, with added loss attributable to broad-spectrum antibiotics. As many as 70 of 73 taxa were decreased in this setting [43], although this does not linearly correlate with clinical outcomes. Next-generation approaches have built upon culture-based studies showing disproportionate loss of commensal organisms from both major families, *Bacteroidetes* and *Firmicutes*, which are typically dominant with concentrations of 10^12^–10^14^ CFU/ml. Disappearance of certain genera among critically ill patients was consistently noted, especially *Faecalibacterium*, *Blautia*, and *Ruminococcus,* and the functional implications are yet to be elucidated from multi-’omic work.

Pathogenic species are thought to encroach on ecological niches created by microbiome disruption [26, 43]. In all reporting studies, organisms which reached metagenomic dominance by any metric, at any body site, predict both colonisation and infectious outcomes. Dominance can *predate* colonisation by as many as 41 days, and nosocomial infection by 3–21 days [35]. By contrast, in no healthy control samples did one pathogen constitute more than 70% of abundant taxa [10].

In critical illness, coincident biodiversity loss and pathogen dominance was first reported by Iapachino who found a massive presence of *Enterococcus* by denatured gel electrophoresis and selective PCR [54]. A decade later, Freedberg et al*.* demonstrated *Enterococcus* dominance by 16S analysis (and colonisation culture) at ICU admission was an independent predictor of infection and death [26]. In this review, up to 80% of studies featured high abundance of *Enterococcus,* associated with in-hospital mortality in 3 studies—where alpha diversity was not—as well as other unfavourable clinical and ecological sequelae (Additional file 1: Table S3).

Moreover, *Enterococcus* has been associated with emergence of drug resistance. An epidemiological study of VRE colonisation found this related to high relative abundance of the genus, and in turn, worst outcomes including mortality within 30 days [55]. While posited as maladaptive ‘overgrowth’, the mechanism by which *Enterococcus* moderates clinical outcomes is elusive and may be antibiotic dependent. Within a cohort exhibiting *Pseudomonas* colonies resistant to carbapenems, piperacillin–tazobactam abolished potentially protective taxa and increased risk of *Enterococcus* dominance [56].

Box 2 Universal features of the critically ill gut in adults and children

**Table:** 

There is a dynamic and dramatic reduction of richness and evenness, with disproportionate loss of beneficial commensal organisms
Overabundance of harmful pathogen organisms accompanies loss of diversity even in antibiotic naïve patients
Loss of biogeography is seen
Ecological patterns cannot be mapped to univariates or disease severity
Organisms known to confer multidrug resistance grow abundant over time, and abundance can precede clinical culture result

### Heterogeneity and microbiome nomenclature is a barrier to consensus

A major challenge for this meta-analysis is the lack of consensus definitions for dysbiosis its core indices for intra- and inter-individual comparison. The recent Strengthening the Organising and Reporting of Microbiome Studies (STORMS) guidelines represent a major step forward in the reporting domain [57].

Heterogeneity is layered in study population, design, sampling and processing methodology, reporting and visualisation. Included studies examine patients admitted to diverse or subspecialist intensive care units and several stratified their results by biomarker, colonisation or other status. Amplicon-based approaches deliver structural taxonomy—conventionally to genus level—upon which function can be inferred. Shotgun metagenomic sequencing delivers subspecies granularity and functional information, however this may obscure hierarchical patterns, with attendant cost and time constraints [9, 58]. Validated methods to adjust for these differences at each pipeline stage are nascent [59, 60].

Common alpha diversity metrics are semi-redundant, and the superior choice of these is not established. Selection of metrics utilised was non-systematic introducing high risk of reporting bias, and in cases only pairwise *p* values were available. Eligible studies traversed clinical, ecological, ‘resistome’, or methodological research domains. Without standardisation, quality concerns regarding presentation of exposure and outcome variables will persist.

Beta diversity is analysed by taxonomically weighted or unweighted measures of similarity or difference, usually with PERMANOVA comparison and visualisation by Principal Component Orthogonal Analysis plot (see Additional file 3: Fig. S7). This has been qualitatively assessed as comparative clustering is not readily leant to aggregation. As authors are increasingly publishing their data to open-access platforms, pooled or network analyses warrant evaluation.

### Limitations

The included studies were small, the largest registering 301 participants [26]. The varied clinical implications of dysbiosis are infrequently reported leading to exclusion of several high-quality ecological studies. A consensus on the optimal methods for interpretation of metagenomic data is still maturing and the major heterogeneity seen in this meta-analysis will not be resolved by larger studies alone. There is an attendant risk of type II error.

Several modulators were not interrogated by this systematic review due to lack of available data and inability to aggregate or transform various exposures and outcomes. The effect of vasopressors, renal replacement therapy, feeding, medications such as proton pump inhibitors, selective digestive decontamination, and mechanical ventilation on gut dysbiosis is unknown.

### Directions

This review prompts collection of species or strain-resolved genomes in future studies, given the frequent mortality association with pathogen overabundance. Large, rigorous longitudinal studies are needed to answer the diversity–disease question in this population, and to interrogate exposures such as antibiotics and artificial feeding (and route). These could be embedded using existing MDR screening procedures or as part of novel trials, such as a salvage FMT for refractory septic shock. Data are currently insufficient to target interventions to subclinical groups, however a randomised adaptive trial design could simultaneously evaluate multiple candidate gut therapies with established safety profiles. An array of research directions are laid out in Table 3.

## Conclusion

This systematic review of early gut dysbiosis identified that larger, harmonised metagenomic studies in both adult and child cohorts are needed. There are few large, rigorous observational studies within the critically ill population with high heterogeneity. Globally, alpha diversity was dynamically reduced in early ICU admission and was not associated with in-hospital mortality. The abundance of taxa such as *Enterococcus* spp. appears to offer greater predictive capacity for important clinical and ecological outcomes.

### Take-home message

1. Alpha diversity was reduced in early ICU admission in adults and children but was not associated with in-hospital mortality (low-certainty evidence).
2. The abundance of taxa such as *Enterococcus* spp. appears to offer greater predictive capacity for important clinical and ecological outcomes.

## Supplementary Information

**Additional file 1: Table S1.** Demographic composition, control group and disease severity measures of studies included in systematic review. **Table S2.** Design and Methodology features of included studies in the systematic review. **Table S3.** Number of studies reporting adverse outcomes associated with increased relative abundance and/or dominance of Enterococcus genus. **Figure S1.** Longitudinal intra-individual trend of early gut microbiome samples, stratified by alpha diversity reporting metric. Described relative to earliest same from same patient. X axis represents number of studies reporting that alpha diversity metric, Y axis lists metrics reported. Bar in blue represents a decrease in alpha diversity. Bar in Orange represents no detectable change in diversity. Grey Bar represents studies which did not report alpha diversity direction of change, if any. **Figure S2.** Alpha diversity lower gut samples at earliest timepoint in ICU admission described relative to non-critically ill controls, stratified by reporting metric. X axis represents percentage of included of studies reporting alpha diversity, Y axis lists metrics reported. Bar in blue represents a decrease in alpha diversity. Bar in Orange represents no detectable change in diversity. **Figure S3.** Distribution of hypervariable regions of interest among 25 of 26 systematic review studies employing amplicon based sequencing. **Figure S4.** Proportion of included studies (%) reporting increased versus decreased variability in microbiome between individuals or over course of ICU admission. **Figure S5.** Proportion of included studies reporting accrual of multi-drug resistant organisms over course of ICU admission. Wedge in dark blue represents confirmed progression of resistance, light blue indicates studies where characteristically multi-resistant organisms increased in relative abundance but resistance was not tested in vitro. Grey represents studies which did not report this phenomenon. **Figure S6.** Taxonomic flux in sepsis. Radar plot representing the number of studies in systematic review reporting enrichment of a typically pathogenic genus among critically ill patients with sepsis or septic shock.

**Additional file 2.** Glossary of Microbiome-related terms.

**Additional file 3. Fig. S7.** Commonly reported metrics of the microbiome in clinical studies. Top left panel represents alpha diversity, a composite index representing richness, eveness and dominance of species within a community. Beta diversity (pink banner panels) represents compositional change between communities within an individual over time or between body sites, or between individuals. Relative, or proportional, and absolute abundance of taxa are depicted in the lower middle panel, together with visualisation of log scale change of species within effect size panel (bottom right).

## Data Availability

Not applicable.
