# Supplementary material for: Lower gut dysbiosis and mortality in acute critical illness: a systematic review and meta-analysis
Source: Intensive Care Med Exp. 2023 Feb 3;11:6. doi: 10.1186/s40635-022-00486-z (PMC9895325; doi:10.1186/s40635-022-00486-z)
Supplement: Supplementary file 1 — Additional file 1: Table S1. Demographic composition, control group and disease severity measures of studies included in systematic review. Table S2. Design and Methodology features of included studies in the systematic review. Table S3. Number of studies reporting adverse outcomes associated with increased relative abundance and/or dominance of Enterococcus genus. Figure S1. Longitudinal intra-individual trend of early gut microbiome samples, stratified by alpha diversity reporting metric. Described relative to earliest same from same patient. X axis represents number of studies reporting that alpha diversity metric, Y axis lists metrics reported. Bar in blue represents a decrease in alpha diversity. Bar in Orange represents no detectable change in diversity. Grey Bar represents studies which did not report alpha diversity direction of change, if any. Figure S2. Alpha diversity lower gut samples at earliest timepoint in ICU admission described relative to non-critically ill controls, stratified by reporting metric. X axis represents percentage of included of studies reporting alpha diversity, Y axis lists metrics reported. Bar in blue represents a decrease in alpha diversity. Bar in Orange represents no detectable change in diversity. Figure S3. Distribution of hypervariable regions of interest among 25 of 26 systematic review studies employing amplicon based sequencing. Figure S4. Proportion of included studies (%) reporting increased versus decreased variability in microbiome between individuals or over course of ICU admission. Figure S5. Proportion of included studies reporting accrual of multi-drug resistant organisms over course of ICU admission. Wedge in dark blue represents confirmed progression of resistance, light blue indicates studies where characteristically multi-resistant organisms increased in relative abundance but resistance was not tested in vitro. Grey represents studies which did not report this phenomenon. Figure S6. Taxonomic flux in sepsis. R [file 40635_2022_486_MOESM1_ESM.docx]

**Additional Materials**

Table S1: Demographic composition, control group and disease severity measures of studies included in systematic review.

| Author/ Date | Number of participants | Mean Age (sd) | Female (%) | Disease Severity | Mean Score | Invasive Ventilation (%) | Number of Controls |
| --- | --- | --- | --- | --- | --- | --- | --- |
| Zaborin 2014 | 14 |  |  | |  |  | 6 |
| Rogers 2016 | 37 | 2.96(2.12) |  | |  |  | 13 |
| Yeh 2016 | 32 | 49.7(na) | 37.5 | APACHE II | 12.6 |  | # |
| McDonald 2016 | 115 | 54.5 (17.0) | 45.0 | APACHE II | 23.7 | 100 | ** |
| Howard 2017 | 12 | 49.0(23.2) | 8.33 | ISS | 34.6 |  | 10^ |
| Lankelma 2017 | 34 | 64.0 (12.1) | 52.9 | APACHEIV | 84.1 | 79.4 | 15 |
| Lamarche 2018 | 34 | 66.6 (10.9) | 41.2 | APACHE II | 25.5 | 100 | 35 |
| Wan 2018 | 15 | 59.7(13.7) | 20.0 | APACHE II | 24.1 |  | 15 |
| Freedberg 2018 | **301** |  |  | **SAPS3** | not specified |  |  |
| Aardema 2018 | 97 | 68(na) | 24.7 | APACHE IV | 48.0 | 100 |  |
| Bansal 2018 | 9 | 65.7(12.1) | 33.3 |  |  |  |  |
| Wijeyesekera 2019 | **60** | **5.86(4.40)** |  | |  |  | 55 |
| Xu 2019 | 98 | 57.0(19.0) | 39.7 | APACHE II | 13.4 | 43.1 | 84 |
| Ravi 2019 | **24** | **54.6 (14.8)** | **20.8** | **SOFA** | **7.4** |  |  |
| Liu 2020 | 64 | 57.71(18.9) | 45.3 | APACHE II | 20.4 |  |  |
| Ojima 2020 | 71 | 62.5(20.4) | 32.4 | APACHE II | 22.9 | 100 |  |
| Burmeister 2020 | 67 | 45.0(2.30) | 34.4 | ISS | 20.9 |  |  |
| Fontaine 2020 | 31 | 52.0(39.3) | 35.5 | SOFA | 8.1 | 100 |  |
| Chernevskaya 2020 | 18 | 50.9(16.4) | 50.0 |  |  | 38.9 |  |
| Agudelo-Ochoa 2020 | 155 | 56.1(18.7) | 46.5 | APACHE-II | 18.1 |  |  |
| Du 2021 | **25** | **3.29(3.07)** | **36** |  |  | **60.0** |  |
| Garcia 2021 | **62** | **63.7(12.9)** | **32.3** |  |  | **27.4** |  |
| Liu 2021 | 20 | 3.60 (1.88) | 20 |  |  |  |  |
| Wei 2021 | 61 | 60.2 (19.0) | 31.2 | APACHE II | 20.4 |  |  |
| Kuo 2021 | 78 |  | 39 | SOFA | 1.5 |  |  |
| Prevel 2022 | **57** | **75(65-79)** |  | **SAPS2** | **69(49-78)** | **56.1** |  |

# American Genome Project

* as well as Human Microbiome Project

**Global Gut Study; mammalian corpse; house surface samples

^ uninjured controls

Table S2. Design and Methodology features of included studies in the systematic review

| **First Author; Date** | **Recruitment Strategy** | **Sample type** | **Metagenomic approach** | **Primers** | **Sequencing Instrument** | **Indexing and key packages** | **Pipeline** |
| --- | --- | --- | --- | --- | --- | --- | --- |
| Zaborin 2014 | Sequential | Stool | 16S | 577F;927R | Illumina | Green genes | QIIME |
| Rogers 2016 | Non-systematic | Stool | 16S | 338F;906R | Roche 454 | Green genes; UCLUST | QIIME 1.8 |
| Yeh 2016 | Non-systematic | Rectal and Stool | 16S | 515F;806R | Illumina MiSeq | Green genes; UCLUST | QIIME |
| McDonald 2016 | Non-systematic | Stool | 16S | nd | Illumina MiSeq | Green Genes 13_8 | QIIME |
| Howard 2017 | Non-systematic | Rectal (DRE) | 16S | nd | Illumina MiSeq | Green genes | QIIME |
| Lankelma 2017 | Randomised | Stool | 16S | 27F;338R | Illumina MiSeq | Silver111; UCLUST | Canoco |
| Lamarche 2018 | Non-systematic | Stool | 16S | nd | Illumina MiSeq | Green genes; UCLUST | QIIME |
| Wan 2018 | Sequential | Stool | 16S | AP221-01 | Illumina MiSeq | Green genes | QIIME; Mothur |
| Freedberg 2018 | Non-systematic | Rectal | 16S | nd | Illumina MiSeq | Green genes | QIIME |
| Aardema 2018 | Sequential | Stool | 16S | 806R;341F | Illumina MiSeq | Illumina | nd |
| Bansal 2018 | Non-systematic | Rectal and Stool | 16S | nd | Illumina MiSeq | USEARCH | QIIME |
| Wijeyesekera 2019 | Sequential | Stool | 16S | nd | Illumina MiSeq | Illumina | SILVA; Mothur |
| Xu 2019 | Non-systematic | Stool | 16S | V4F; V4R | Illumina HiSeq | Green genes | QIIME |
| Ravi 2019 | Sequential | Stool | Shotgun | Universal Bacterial Primers | Nextseq550 | Illumina Nextera X T | MetaPhlAn2 |
| Liu 2020 | Non-systematic | Stool | 16S | nd | Illumina MiSeq | UCLUST | nd |
| Ojima 2020 | Sequential | Rectal | 16S | nd | Illumina MiSeq | Green genes | QIIME 2 |
| Burmeister 2020 | Non-systematic | Rectal (DRE) | 16S | F27;R355 | Illumina MiSeq | Green genes | QIIME |
| Fontaine 2020 | Non-systematic | Stool | 16S | nd | Illumina MiSeq | Illumina | Shaman |
| Chernevskaya 2020 | Non-systematic | Stool | 16S | nd | Iontorrent PGM | Green genes | QIIME |
| Agudelo-Ochoa 2020 | Sequential | Rectal | 16S | S-D-Bact-03410B-S-17; S-D-Bact-0785-aA-21 | Illumina MiSeq | UCLUST; USEARCH | QIIME; Mothur |
| Du 2021 | Non-systematic | Rectal | 16S | 515F; 806R | Illumina HiSeq | UCLUST; Green Genes | QIIME |
| Garcia 2021 | Non-systematic | Rectal and Stool | 16S | nd | Illumina MiSeq | Illumina | QIIME 2 |
| Liu 2021 | Non-systematic | Stool | 16S | 515F;806R | Illumina HiSeq | Illumina | QIIME 2; Mothur |
| Wei 2021 | Sequential | Rectal and Stool | 16S; 18S; ITS2 | V4: 515F-806R, 18S V4: 528F-706R, 18S V9: 1380F-1510R | Illumina HiSeq | Illumina | UPARSE; SILVA; MUSCLE |
| Kuo 2021 | Sequential | Stool | 16S | HMP primer or illumina primers | Illumina (not specified) | Green genes; DADA2; phyloseq | QIIME 2 |
| Prevel 2022 | Sequential | Rectal | 16S; 18S; ITS2 | nd | Illumina MiSeq | vegan; phyloseq | DADA2 |

Nd=no data. Rectal refers to rectal swab unless otherwise stated. DRE=digital rectal exam. Contamination protocol, biomass measure, and risk of skew or undersampling infrequently reported. None of the included studies used Oxford Nanopore or Pacific Biosciences technology.

Table S3. Number of studies reporting adverse outcomes associated with increased relative abundance and/or dominance of Enterococcus genus.

| **Adverse Outcome** | **Number of Reporting Studies** |
| --- | --- |
| *Clinical* |  |
| Risk of Infection | 2 |
| Mortality | 3 |
| Increased Inflammatory Markers | 1 |
| ICU LOS | 1 |
| *Ecological* |  |
| Decreased Diversity | 3 |

Note: Enteroccocus was detected by abundance alone in 17 studies, or paired with colonisation status (3 studies). It was enriched in all studies reporting this specifically (n=20).

Figure S1. Longitudinal intra-individual trend of early gut microbiome samples, stratified by alpha diversity reporting metric. Described relative to earliest same from same patient. X axis represents number of studies reporting that alpha diversity metric, Y axis lists metrics reported. Bar in blue represents a decrease in alpha diversity. Bar in Orange represents no detectable change in diversity. Grey Bar represents studies which did not report alpha diversity direction of change, if any.

Figure S2. Alpha diversity lower gut samples at earliest timepoint in ICU admission described relative to non-critically ill controls, stratified by reporting metric. X axis represents percentage of included of studies reporting alpha diversity, Y axis lists metrics reported. Bar in blue represents a decrease in alpha diversity. Bar in Orange represents no detectable change in diversity.

Figure S3. Distribution of hypervariable regions of interest among 25 of 26 systematic review studies employing amplicon based sequencing.

Figure S4. Proportion of included studies (%) reporting increased versus decreased variability in microbiome between individuals or over course of ICU admission.

Figure S5. Proportion of included studies reporting accrual of multi-drug resistant organisms over course of ICU admission. Wedge in dark blue represents confirmed progression of resistance, light blue indicates studies where characteristically multi-resistant organisms increased in relative abundance but resistance was not tested in-vitro. Grey represents studies which did not report this phenomenon.

Figure S6. Taxonomic flux in sepsis. Radar plot representing the number of studies in systematic review reporting enrichment of a typically pathogenic genus among critically ill patients with sepsis or septic shock.
