# Supplementary material for: Lower gut dysbiosis and mortality in acute critical illness: a systematic review and meta-analysis
Source: Intensive Care Med Exp. 2023 Feb 3;11:6. doi: 10.1186/s40635-022-00486-z (PMC9895325; doi:10.1186/s40635-022-00486-z)
Supplement: Supplementary file 2 — Additional file 2. Glossary of Microbiome-related terms. [file 40635_2022_486_MOESM2_ESM.docx]

Additional Material: Glossary of Microbiome-related terms

| **Term** | **Definition** | **Reference** |
| --- | --- | --- |
| 16s rDNA | 16S ribosomal subunit gene, unique to prokaryotic cells, with highly preserved sequence and hypervariable regions, which are amplified and used as markers for bacterial identification. Also referred to as 16S rDNA. | Kitsios et al^1^ |
| Abundance | Prevalence of a particular taxonomic group in a microbial community. Expressed in relative (proportional) or absolute abundance, usually by amplicon-based or selective polymerase chain reaction respectively. | Kitsios et al^1^ |
| Alpha Diversity | Within-sample taxonomic diversity (including richness and eveness) as a summary statistic of a single population. Often expressed as (inverse) Shannon or Simpson's indices. | Szychowiak et al^2^ |
| Amplicon-Based Sequencing | Amplification with polymerase chain reaction and sequencing of specific markers, usually relating to phylogenetically hyper-variable regions | Kitsios et al^1^ |
| Antibiosis | Microbiome changes attributable to administration of antimicrobials of any kind. | Tosh et al^3^ |
| Berge-Parker Dominance | Emergence of a single, overtly abundant taxonomic group. Often expressed as Berge-Parker ominance.proportional abundance of the most abundant type | Berger^4^ |
| Beta Diversity | Between-sample taxonomic diversity describing absolute or relative taxonomic overlap between samples. | Szychowiak et al^2^ |
| Richness | Number of taxonomic groups in a microbial community. Often expressed by Chao richness. | Chao^5^ |
| Commensal microbiota | Microbes that provide benefits to the (human) host without being affected by it. | Baquero^6^ |
| Commensalism | Relationship between two organisms in which one benefits and the other is not affected. | Baquero^6^ |
| Community Structure | Taxonomic composition of a microbial community. | Petersen et al^7^ |
| Culturomics | Method for analyzing bacterial composition of complex samples such as human gut, based on extensive culture media and atmosphere combinations. | Szychowiak et al^2^ |
| Dysbiosis | perturbations to the structure of complex commensal communities; Alteration in the normal composition of the microbiota; can be associated with disease | Petersen et al^7^ |
| Eveness | Relative abundance of different taxonomic groups (Often expressed by Pielou eveness). | Pielou^8^ |
| Functional diversity | Variation of traits between organisms within an ecological unit. | Escalas et al ^9^ |
| Library | Collection of biological DNA fragments prepared for sequencing. | Paulsen et al^10^ |
| Metabolome | Total metabolite pool | Kitsios et al^1^ |
| Metagenome | The collective genomes of members present in a microbial community. Functional metagenomics refers to computational or experimental analysis of a microbial community with respect to the molecular activities of its composite genome. | Kitsios et al^1^ |
| Meta'omics | The study of the total content of a community of microbiota in terms of metagenome, metatranscriptome, metaproteome and metabolome. | Syzchowiak et al^2^ |
| Metaproteome | Entire protein complement. | Kitsios et al^1^ |
| Metatranscriptome | Complete set of RNA transcribed from microbial environments. | Kitsios et al^1^ |
| Microbiome | The totality of human (or other host) body's micro-organisms (including bacteria, viruses, fungi, archaea, protozoa), their genomes and molecular products, and the surrounding environmental conditions. | Kitsios et al^1^ |
| Microbiota | The complex of microorganisms (bacteria, parasites, viruses, fungi) that colonize a host or a habitat | Backhed ^11^ |
| Mutualism | Relationship between two organisms in which both benefit | Szychowiak et al^2^ |
| Next Generation Sequencing | A culture-independent molecular sequencing method. Differs from Sanger sequencing. | Wolffe^12^ |
| Operational Taxonomic Unit | Clusters of sequences sharing a minimal identity (e.g., 97% is commonly used in 16S studies), referring to a taxonomic group. These clusters and the respective number of reads within are an estimation of the abundance of different taxa in samples | Szychowiak et al^2^ |
| Pangenome | The entire gene set of a species | Langille et al ^13^ |
| Parasitism | One organism benefits at the expense of another | Kitsios et al^1^ |
| Shannon Index | An entropy measure quantifying the uncertainty of selecting a type. In ecology, it is a summary informational statistic of alpha diversity representing the weighted geometric mean of the proportional abundances of all types in the sample. It will be increased with increasing richness and eveness within a community. | Berg et al^14^ |
| Simpson’s Index | A dominance statistic used to measure alpha diversity, representing the weighted arithmetic mean. More reliant on common species than shannon index. | Berg et al^14^ |
| Symbiosis | Broader category encompassing relationships that are commensal, mutualistic or parasitic | Szychowiak et al^2^ |
| Taxon (plural taxa) | A grouping of microbes defined by the degree of genetic identity, often >75% for phylum, >80% for class, >85% for order, >90% for family, >95% for genus and >97% for species. | Szychowiak et al^2^ |
| Taxonomy | Microbial classification into ordered taxa (categories) | Szychowiak et al^2^ |
| Whole metagenome shotgun sequencing | Sequencing of short, random DNA/RNA fragments in an undirected whole-genome fashion | Berg et al^14^ |

References

1 Kitsios, G. D. *et al.* Dysbiosis in the intensive care unit: Microbiome science coming to the bedside. *J Crit Care* **38**, 84-91 (2017). <https://doi.org:10.1016/j.jcrc.2016.09.029>

2 Szychowiak, P., Villageois-Tran, K., Patrier, J., Timsit, J. F. & Ruppe, E. The role of the microbiota in the management of intensive care patients. *Ann Intensive Care* **12**, 3 (2022). <https://doi.org:10.1186/s13613-021-00976-5>

3 Tosh, P. K. & McDonald, L. C. Infection control in the multidrug-resistant era: tending the human microbiome. *Clin Infect Dis* **54**, 707-713 (2012). <https://doi.org:10.1093/cid/cir899>

4 Berger, W. H. & Parker, F. L. Diversity of planktonic foraminifera in deep-sea sediments. *Science* **168**, 1345-1347 (1970).

5 Chao, A. Nonparametric estimation of the number of classes in a population. *Scandinavian Journal of statistics*, 265-270 (1984).

6 Baquero, F., Coque, T. M. & de la Cruz, F. Ecology and evolution as targets: the need for novel eco-evo drugs and strategies to fight antibiotic resistance. *Antimicrob Agents Chemother* **55**, 3649-3660 (2011). <https://doi.org:10.1128/AAC.00013-11>

7 Petersen, C. & Round, J. L. Defining dysbiosis and its influence on host immunity and disease. *Cell Microbiol* **16**, 1024-1033 (2014). <https://doi.org:10.1111/cmi.12308>

8 Pielou, E. The measurement of diversity in different types of biological collections. *Journal of Theoretical Biology* **15**, 177-177 (1967).

9 Escalas, A. *et al.* Microbial functional diversity: From concepts to applications. *Ecol Evol* **9**, 12000-12016 (2019). <https://doi.org:10.1002/ece3.5670>

10 Poulsen, C. S., Ekstrøm, C. T., Aarestrup, F. M. & Pamp, S. J. Library Preparation and Sequencing Platform Introduce Bias in Metagenomic-Based Characterizations of Microbiomes. *Microbiology Spectrum* **10**, e00090-00022 (2022).

11 Bäckhed, F. *et al.* Defining a healthy human gut microbiome: current concepts, future directions, and clinical applications. *Cell host & microbe* **12**, 611-622 (2012).

12 Wolff, N. S., Hugenholtz, F. & Wiersinga, W. J. The emerging role of the microbiota in the ICU. *Crit Care* **22**, 78 (2018). <https://doi.org:10.1186/s13054-018-1999-8>

13 Langille, M. G. *et al.* Predictive functional profiling of microbial communities using 16S rRNA marker gene sequences. *Nat Biotechnol* **31**, 814-821 (2013). <https://doi.org:10.1038/nbt.2676>

14 Berg, G. *et al.* Microbiome definition re-visited: old concepts and new challenges. *Microbiome* **8**, 103 (2020). <https://doi.org:10.1186/s40168-020-00875-0>
