# Supplementary figures and images for: Lower gut dysbiosis and mortality in acute critical illness: a systematic review and meta-analysis
Source: Intensive Care Med Exp. 2023 Feb 3;11:6. doi: 10.1186/s40635-022-00486-z (PMC9895325; doi:10.1186/s40635-022-00486-z)

# Metrics of the microbiome

INTRA-INDIVIDUAL

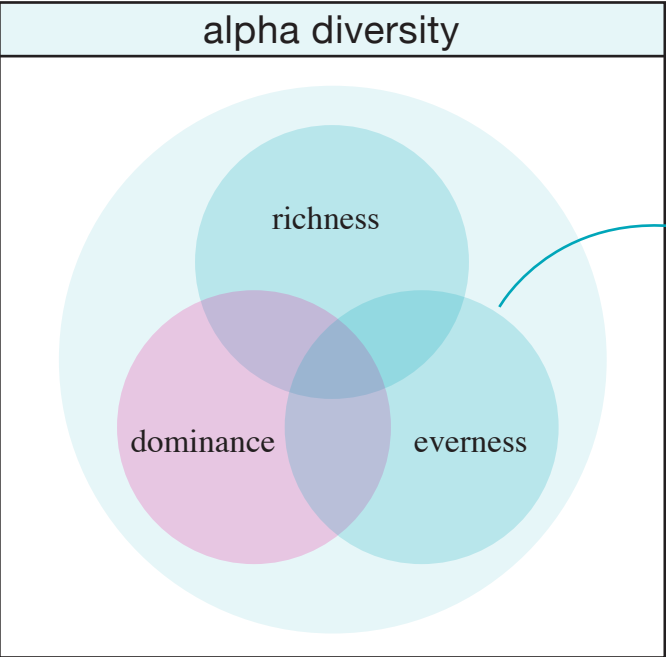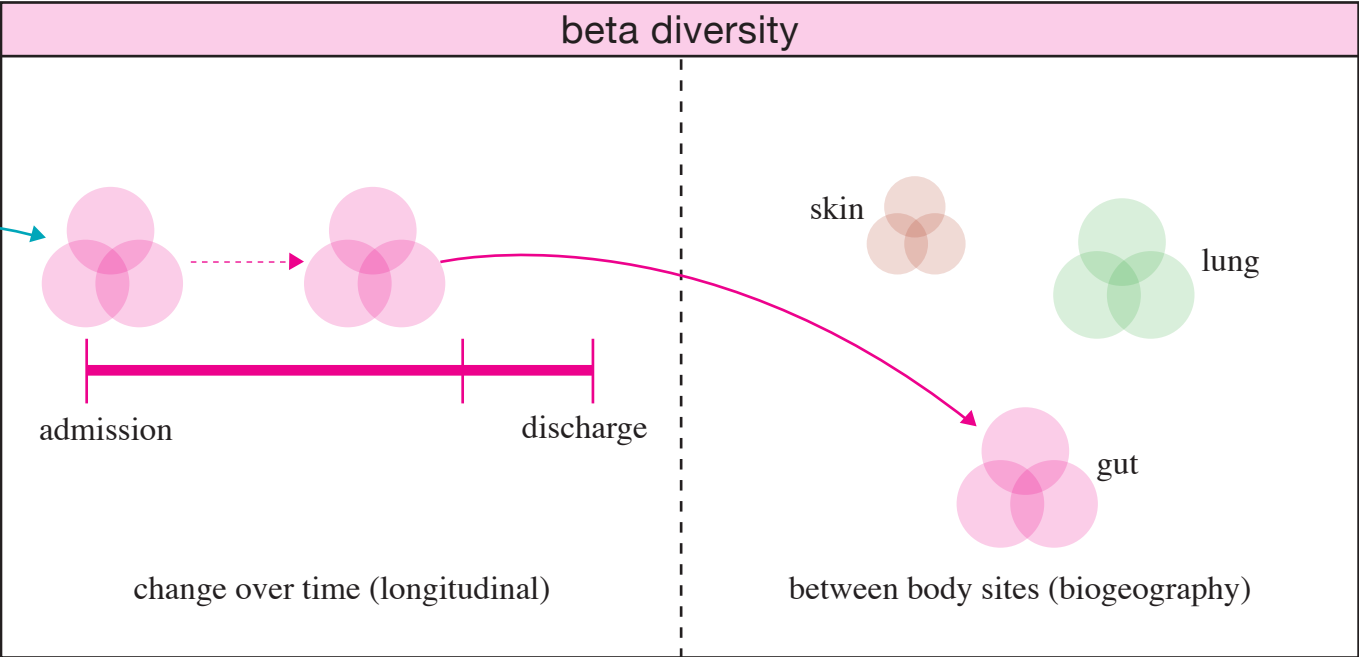

BETWEEN INDIVIDUAL

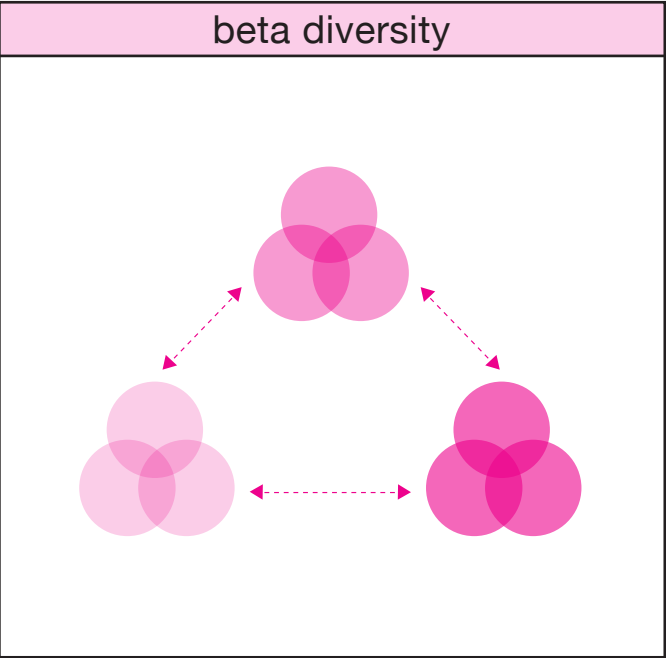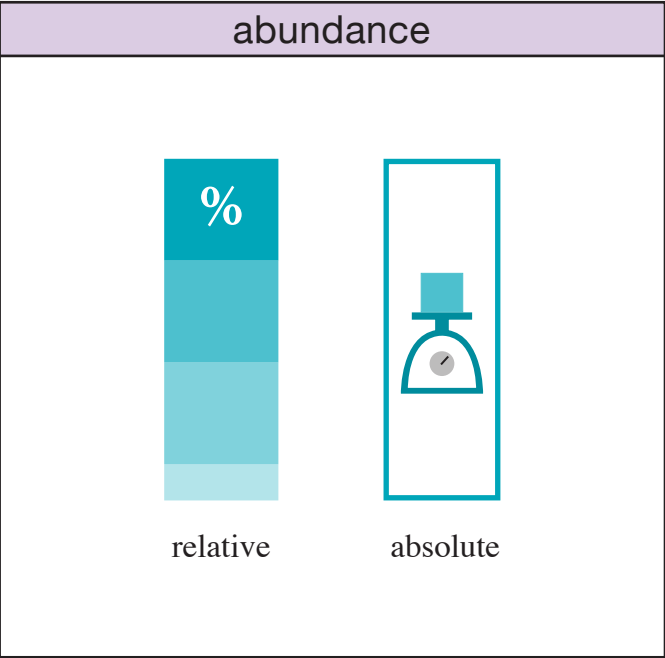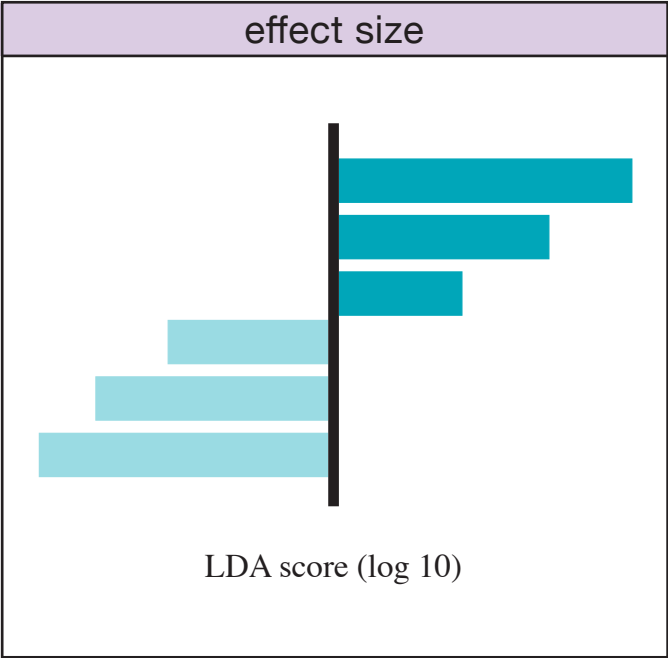

Supplement: Supplementary file 3 — Additional file 3. Fig. S7. Commonly reported metrics of the microbiome in clinical studies. Top left panel represents alpha diversity, a composite index representing richness, eveness and dominance of species within a community. Beta diversity (pink banner panels) represents compositional change between communities within an individual over time or between body sites, or between individuals. Relative, or proportional, and absolute abundance of taxa are depicted in the lower middle panel, together with visualisation of log scale change of species within effect size panel (bottom right). [file 40635_2022_486_MOESM3_ESM.pdf]
